# Contextual Correlates of Cognitive Aging in Black Older Adults: Examining the Neighborhood

**DOI:** 10.1093/geroni/igab046.185

**Published:** 2021-12-17

**Authors:** Ketlyne Sol, Tanisha Hill-Jarrrett

**Affiliations:** 1 University of Michigan, Ann Arbor, Michigan, United States; 2 University Of South Florida Morsani College Of Medicine, Tampa, Florida, United States

## Abstract

Black older adults have a unique history that includes enslavement and legalized segregation. This history shapes the present-day experiences of older Blacks, in part, through the neighborhoods in which they live. The neighborhood is a reflection of both the physical and social contexts, and reflects the most natural and intimate context through which a person experiences life. Combined, the unique history and neighborhoods of Black older adults may contribute to their disproportionately experiencing impairments in cognitive function in older age. There is growing interest in how lived experiences across the life course affect cognitive trajectory and, ultimately, cognitive outcomes of older Black adults. In this presentation, we will review recent literature on psychosocial and physical contextual factors and their influence on cognitive aging in older blacks through the lens of the neighborhood.

